# Application of Biostimulant in Seeds and Soil on Three Chickpea Varieties: Impacts on Germination, Vegetative Development, and Bacterial Facilitation of Nitrogen and Phosphorus

**DOI:** 10.3390/life14010148

**Published:** 2024-01-19

**Authors:** Elisa Gómez, Alejandro Alonso, Jorge Sánchez, Pedro Muñoz, José Marín, David Mostaza-Colado, Pedro V. Mauri

**Affiliations:** 1 Instituto Madrileño de Investigación y Desarrollo Rural, Agrario y Alimentario (IMIDRA) Finca El Encín, Autovía A-2. Km 38,200, 28805 Alcalá de Henares, Spain; elisamercedes.gomez@madrid.org (E.G.); rafaelalejandro.alonso@madrid.org (A.A.); jorge.sanchez.hernandez@madrid.org (J.S.); pedro.munoz@madrid.org (P.M.); david.mostaza@madrid.org (D.M.-C.); 2 Área Verde MG Projects S.L., C/Oña 43, Bajo, 28050 Madrid, Spain; jmarin@areaverde.es

**Keywords:** *Cicer arietinum* L., Amelia (AM), IMIDRA 10 (IM), Blanco Sinaloa (BS), biostimulant, days after sowing (DAS), germination percentage (GP), nitrogen-fixing bacteria (NFB), phosphate-solubilising bacteria (PSB)

## Abstract

Chickpeas (*Cicer arietinum* L.) are a valuable legume crop due to their nutritional value. To maintain chickpea productivity and avoid the adverse effects of climate change on soil and plant processes, it is crucial to address demand. Achieving this necessitates implementing sustainable agricultural practices incorporating the use of biostimulants, adaptable crops for arid conditions, as well as pest and disease-resistant crops that are sustainable over time. Three varieties of chickpeas were analysed to determine the effect of two different biostimulant application methods on both germination and vegetative growth. Possible effects due to location were also examined by conducting tests at two different sites. Significant variations in biostimulant response were evident only during the germination period, but not during the vegetative development stage, where the observed statistical differences were influenced more by the location or variety of chickpeas employed. Furthermore, this study examined the effect of biostimulants on nutrient cycling within the soil–plant microbiota system. Nitrogen-fixing bacteria (NFB) are present in the soil of chickpea crops at an order of magnitude of 10^7^ CFU/g DS. Additionally, an average concentration of 10^6^ CFU/g DS of phosphorus-mobilising bacteria was observed. Applying biostimulants (BioE) to seeds resulted in a successful germination percentage (GP) for both Amelia (AM) and IMIDRA 10 (IM) varieties.

## 1. Introduction

Due to climate change, crops are impacted by drought, resulting in biochemical and physiological consequences for plants. These effects influence parameters including germination, vitality, and yield, in addition to seed composition and nutrition [1,2].

The Food and Agriculture Organization of the United Nations (FAO) strives to advocate for policies and practices that endorse the integration of agriculture with the productive sector and ensure accountable management and prolonged availability of natural resources [3]. The necessity of sustainable agriculture is now apparent, as predicted in the 2030 Agenda for Sustainable Development [4].

Contemporary agriculture is changing to establish more sustainable crops that are adaptable to climate change without compromising final yield. The utilisation of biostimulants is one of the suggested methods to meet these challenges [5].

Crop yields vary depending on the crop variety, soil, climate conditions, and pest and disease management techniques [5]. It is crucial to employ technologies like biostimulants that enhance germination success and improve vegetative development to boost crop efficiency and address these challenges. It is advantageous to implement agricultural methods that prevent reproductive stages coinciding with periods of high temperature and water stress and to cultivate varieties with earlier sowing dates to adapt to climate change [6,7]. The objective is to modernise chickpea production using novel technologies to enhance yields and economic viability while transferring the resulting knowledge to agricultural practitioners to bridge the technology gap. If the results are favourable, this approach promises higher yields and reduced resource consumption, leading to more efficient harvesting [1].

Plant biostimulants are defined in Regulation (EU) 2019/1009 as “an agricultural product regulated by the European Union, designed to improve nutritional processes in plants and their rhizosphere. The purpose is solely to enhance one or more of the following plant characteristics: (i) nutrient usage efficiency, (ii) tolerance to abiotic stress, (iii) quality traits, or (iv) limited nutrient availability in the soil or rhizosphere. The product’s nutrient content is not a determining factor in its function” [8].

According to their composition, plant biostimulants are categorised into non-microbial and microbial classifications [9]. The former category includes chitosan [10], humic and fulvic acids [11], protein hydrolysates [12], phosphites [13], seaweed extracts [14], and silicon [15]. In the second category, there are arbuscular mycorrhizal fungi (AMF) [16], plant-growth-promoting rhizobacteria (PGPR) [17], and *Trichoderma* spp. [18].

The significance of chickpeas and lentils (*Lens culinaris* Medik.), much like other grain legumes, lies in their provision of vegetable protein that complements the carbohydrate content derived from cereals. Chickpeas are recognised as one of the most vital pulses globally owing to their high nutritional value. This drought-resistant legume is capable of living in a symbiotic association with *Rhizobium* sp. bacteria and microorganisms that fix atmospheric nitrogen in the plant and soil. This helps to control soil erosion and utilise water efficiently. Additionally, because it fixes atmospheric nitrogen and requires no potassium, except for neutralising the effect of limestone, it reduces the usage of inputs and aids in mitigating the consequences of climate change. It is also abundant in proteins, carbohydrates, minerals, starch, and lipids, notably oleic and linoleic unsaturated fatty acids, and has minimal levels of cholesterol [19].

Grown in rotation with cereals, its production costs are minimal, and it is well suited to the arid conditions of Spain [20]. The most prevalent disease that affects this crop is chickpea rabies, which is induced by the fungus *Ascochyta rabiei* Pass. Dry rot poses a common issue in chickpea cultivation, which may be caused by various pathogens, amongst which the primary one is *Fusarium oxysporum* f. sp. *ciceri*. There are cultivars resistant to common diseases that will be used in this study. Such tolerant cultivars are preferred because chemical treatment is not cost effective. There are no characteristic weeds specific to chickpea cultivation, although certain genera cause great impact, such as *Poligonum*, *Convolvulus*, *Euphorbia*, *Avena*, *Amaranthus*, *Galium*, *Phalaris*, etc., which are prevalent in the central part of the peninsula [20]. It is hypothesised that biostimulants also promote weed growth in species more competitive than chickpeas [21].

Chickpea cultivation faces myriad challenges, with the availability of nitrogen (N) and phosphorus (P) being two critical factors that limit its yield and quality. As agriculture endeavours to strike a balance between food production and environmental sustainability, it becomes imperative to explore strategies that enhance the efficient use of these essential nutrients [22,23]. Nitrogen-fixing bacteria (NFB) and phosphorus-solubilising bacteria (PSB) are pivotal components of sustainable agricultural systems [24] due to their capacity to enhance N and P availability to host plants. NFB, through symbiosis with legumes, can convert atmospheric nitrogen into plant-assimilable forms [25], whereas PSB can release immobilised phosphorus in the soil, rendering it accessible to plant roots. Furthermore, this PGPB (plant-growth-promoting bacteria) stimulates plants to produce a variety of plant growth hormones, including auxins, gibberellins, cytokinins, and ethylene [26].

The primary aim of this study was to examine the impact of two biostimulant application methods on the germination of three types of chickpeas in open fields. This research expanded on earlier work carried out by our research team and utilised our previously acquired observations on the biostimulant type [21] and the mechanical seeder approach [27], as well as biostimulant dosages [1,2,21,27] using three chickpea varieties [27].

Our previous publication [21] is a result of research on chickpea technification. The study focused on the variety Amelia (AM), registered by IMIDRA, and was conducted in both greenhouse and field conditions. The first trial involved the use of varying doses of a biostimulant mixture, and the study evaluated the germination power and vegetative development. After determining the optimal dose, we planned field sowing to test the biostimulant application. The seed was surrounded in a simple and farmer-friendly way that was compatible with traditional agricultural machinery. Our preliminary conclusion was that the biostimulant was successful in the greenhouse at low concentrations. However, in the field, it did not perform as well and appeared to promote weed growth.

Our next publication [27] aimed to investigate new biostimulant formulas both together and separately in three chickpea varieties (AM, Blanco Sinaloa (BS), and IMIDRA 10 (IM)), building on the results of the previous study [21]. The experiment was conducted in a greenhouse using commercial substrate due to the autumn season, which made field testing unfeasible. The objective of that trial was to select the optimal biostimulant and chickpea variety behaviour. It was observed that the positive effect was only evident in the BS variety.

This study is an extension of our previous research, with a particular emphasis on examining germination success and vegetative development under open-field settings, which was measured through plant length (cm) and node quantity over a 48-day period post-showing. The experimental design of this study allowed for the acquisition of previously unavailable data. Furthermore, data from two new locations not previously explored by our team in open-field environments were gathered. Additionally, the analysis was extended to investigate the impact of the biostimulant and the method of its application on the soil–plant–microbiota environment by studying both the NFB and PSB present in the control soil and the one treated with biostimulant. Finally, we assessed the success of germination and vegetative development of different chickpea varieties: (1) AM, (2) BS, and (3) IM. These were studied in the open field using biostimulants in real environmental and temporal conditions.

The proposed research hypothesis posits a positive influence of biostimulant application on various chickpea cultivars concerning germination and vegetative growth. This was compared across different locations and days after sowing (DAS). Moreover, the presence of NFB and PSB in the soil was examined after the different treatments and locations.

The null hypothesis was that biostimulant application would not impact germination, vegetative development, or nitrogen-fixing or phosphorus-solubilising bacteria.

## 2. Materials and Methods

### 2.1. Plant Material

Three varieties of chickpeas, named Amelia (AM), IMIDRA 10 (IM), and Blanco Sinaloa (BS), were analysed in this study. Both AM and IM are rainfed crops, while BS needs irrigation. These rainfed varieties were developed from diverse research projects carried out at the Instituto Madrileño de Investigación y Desarrollo Rural, Agrario y Alimentario (IMIDRA) [20]. These chickpeas were carefully selected from various crosses of adaptable varieties exhibiting high resistance to the soil and climate conditions in the region, especially drought. They display resistance to fungal diseases, such as *Fusarium oxysporum* f sp. *ciceri*. AM, a desi-type variety [28], is sown in February or March and is well-suited to dry soils. It displays excellent resistance to lodging and fungal diseases, like rabies (*Ascochyta rabiei* Pass.). The Kabuli-type IM variety [28,29,30] bears a resemblance to AM, possessing certain similar traits, such as elevated productivity and pest resistance. However, IM contains less phytic acid and has a protein digestibility index of 96% [31]. The Kabuli white chickpea variety BS [30] originates from the agricultural regions in central and northwestern Mexico. These chickpeas are irrigated during growth and are recognised for their significant size, high yield, and resistance to powdery mildew, which is a common fungal disease caused by *Erysiphe* spp.

### 2.2. Biostimulant

The biostimulant used in this study (BioE) comprises a commercial blend of endomycorrhizal fungi, such as *Glomus intraradices*, and other beneficial fungi, including *Trichoderma virens*, *T. harzianum*, *T. reesei*, and *T. viride*, sold as a solid powder (referred to as A) and plant-growth-promoting rhizobacteria (PGPB), sold as another powder (referred to as B). It consists of *Azospirillum brasilense*, *Azotobacter chroococcum*, *Bacillus megaterium*, and *Pseudomonas fluorescens*, as well as vitamins, such as biotin (B7), folic acid (B9), B2, B3, B6, B12, carbon (C), potassium (K), amino acids from protein hydrolysate, soluble extract of *Ascophyllum nodosum*, and *Yucca schidigera*. The manufacturer’s recommended equal weight of powders A and B was used. The diluent utilised was a molasses base derived from plant extracts and seaweed. We mixed powders A and B with the diluent (16.6 g of powder A + 16.6 g of powder B per 100 mL of diluent) following the instructions specified in the manual to create the biostimulant named BioE.

Finally, a concentration of 0.01 L of BioE/kg of chickpea was utilised in conjunction with a control group where no product was applied (control). Two methods of biostimulant application were employed: (1) impregnation of 1 mL of liquid biostimulant per 100 g of seed 24 h before sowing (BioE), and (2) direct application of the biostimulant onto the soil immediately after sowing, thus ensuring its percolation into the soil and around the seed through light irrigation (BioE soil). In the first case (BioE), the tank was shaken twenty times to evenly distribute the biostimulant over the chickpea surface. The seeds soaked in this manner were then placed in 120 mm diameter Petri dishes and left uncovered to prevent excess moisture in the seeds [2,21,27]. The biostimulant selection and dosage for these experiments relied on several years of prior studies conducted by the IMIDRA research group. We applied the most favourable results in each case [1,2,21,27,31].

### 2.3. Field Trial Design

**Figure 1:**
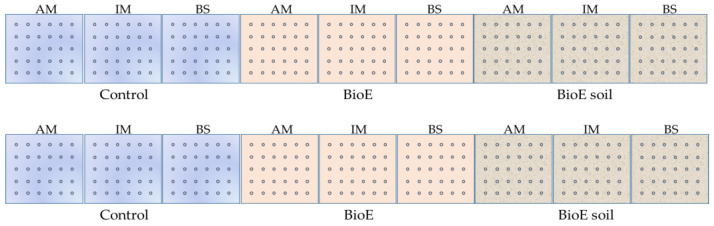
Experimental design of two 9 m^2^ plots (A **up**, B **down**), both subdivided into 9 × 1 m^2^ subplots. Plots A and B were separated by 680 m in an open-field experiment.

The experiment was conducted at IMIDRA, El Encín, within the municipality of Alcalá de Henares, Madrid, Spain (40°31′17″ N; 3°17′21″ W), and it was replicated in two plots, Plot A and Plot B, located 680 m apart in an open field. This study was limited by the operational needs of our research centre, which restricted the space available for plot installation. Thus, it was decided to select two new locations and two dates that were suitable for the available space and field staff. Also, as crop rotation is necessary to avoid planting the same crop in the same soil in consecutive years, this requires the use of different plots. The size of each of the plots was 9 m^2^ (9 × 1 m^2^ length × wide, respectively), which were further subdivided into 9 subplots, each of which was 1 m^2^ (1 × 1 m^2^ length × wide, respectively). To avoid soil contamination across treatments of biostimulant, we carried out a split-plot design (Figure 1).

Thus, the first three subplots were assigned to the control, the three intermediates were assigned to BioE, and the last three were assigned to BioE soil. Within the three subplots of the same biostimulant treatments, the varieties were assigned in the same order. In each of the 1 m^2^ subplots, 30 chickpea seeds were sown by hand at a depth of 5 cm with the help of a seed frame measuring 80 × 75 cm (5 rows × 6 columns). The lateral spacing between seeds was 15 cm, with a frontal spacing of 20 cm. Each experimental unit varied in terms of the administered dosage, the specific type of biostimulant treatment, and the cultivar being examined. Following the initial sowing stage, protective netting was implemented to prevent any potential harm caused by birds or other animals [2,21,27].

Although chickpea is a rainfed crop, the experimental area in the central region of the Iberian Peninsula experienced unfavourable climatic conditions, resulting in a lack of rainwater during the winter and mid-spring seasons. Consequently, the biostimulant treatment and occasional irrigation were necessary to support plant growth.

Data were collected at various times, including 17 days after sowing (DAS), 24 DAS, 33 DAS, 40 DAS, and 47 DAS (3, 10, 19, and 24 April 2023) for Plot A, and 16 DAS, 23 DAS, 34 DAS, 41 DAS, and 48 DAS (21 and 28 April and 9, 16, and 23 May 2023) for Plot B. The data comprised counts of germinated plants in comparison to the number of seeds sown for each variety and treatment. Additionally, measurements of plant length in cm and number of nodes were taken from 10 randomly selected plants of each variety and treatment starting from 23 DAS. 

### 2.4. Soil

This trial took place on the fine clayey soils of subgroup Xerochrept alphic-vertic, with a pH of 8.4, in Plot A, and Xerochrept alphic-fluventic, with a pH of 7.2, in Plot B [32]. These soils display heterogeneity, with their current composition resulting from both climatic and anthropogenic conditioning factors, such as earth movements, natural watercourse diversion, and saline water irrigation. The region has a xeric regime, with a transition between alluvial terraces and the land closest to the river, with a clear fluvial influence and clay translocation processes [32].

Soil samples were collected from sites A and B and analysed for nitrogen (N), soil organic matter (SOM), phosphorus (P), and potassium (K) using the official analysis methods. The results obtained for the studied plots are in Table 1. To investigate the impact of the biostimulant on the system encompassing soil, plant, and microorganisms, soil samples were collected from both sites (Plots A and B) when the flowering was completed. The samples were categorised as either untreated soil (control), soil treated with the biostimulant applied to the seed (BioE), or soil treated with the biostimulant applied directly to the soil (BioE soil). The same type of biostimulant was applied to both plots. Triplicate soil samples of 100 g each were collected for each treatment (control, BioE, and BioE soil) in each experimental plot. The 3 sub-samples of 100 g were homogenised to create a single representative sample per treatment.

### 2.5. Bacterial Counts

For the analysis of viable bacteria in the soil, Ashby culture media [33] were employed to detect atmospheric nitrogen-fixing bacteria (NFB). The components of the medium are briefly detailed as follows: glucose 2.0%, CaCO_3_ 0.5%, K_2_HPO_4_ 0.02%, MgSO_4_ 0.02%, NaCl 0.02%, K_2_SO_4_ 0.01%, and bacteriological agar 1.5%. The pH of the medium was adjusted to 7.4. 

For the enumeration of phosphate-solubilising bacteria (PSB), the National Botanical Research Institute’s Phosphate solid media (NBRIP) was employed. The phosphate solubilisation ability was determined through plate assay [34] Briefly: glucose 1.0%, Ca_3_(PO_4_)_2_ 0.5%, MgCl_2_·6H_2_O 0.5%, (NH_4_)_2_SO_4_ 0.01%, MgSO_4_·7H_2_O 0.025%, KCl 0.02%, and bacteriological agar 1.5%. The pH of the medium should be adjusted to 7.0.

The Most Probable Number (MPN) technique [35] was employed to quantify soil bacteria. To accomplish this, decimal dilutions were prepared in a sterile saline solution of 0.9% (*w*/*v*). The initial dilution involved mixing 10.00 g of soil with 90 mL of sterile saline solution. Afterward, the mixture was homogenised using an orbital shaker operating at 120 rpm while maintaining a temperature of (28.0 ± 0.5) °C for 60 min. A series of dilutions spanning from 10^3^ to 10^5^ was established. For each decimal dilution, 5 sets of 5 µL each were inoculated. Subsequently, these culture media were subjected to incubation at (32.0 ± 1.0) °C for a period of 2 weeks.

### 2.6. Variables Studied

The main objective of this research was to evaluate the influence of biostimulant application on several parameters, such as GP, measured as (number of germinated plants/number of sown seeds) × 100, plant vegetative growth, measured in plant length (cm), and node number, observed on different days after sowing (DAS) in an open-field design assay. In addition, the effect of the biostimulant on the soil microbiome was analysed by monitoring plant-growth-promoting rhizobacteria (PGPR) by focusing on nitrogen-fixing bacteria (NFB) and phosphate-solubilising bacteria (PSB). The experiment was conducted in two different locations (Plots A and B) using three different methods of biostimulant application: zero (control), on seed (BioE), and in soil immediately after sowing (BioE soil).

### 2.7. Statistical Analysis

In every analysis, DAS was included as a covariate. For the full set of data, we tested the main effects of treatment, variety, and location without interactions. The model failed to converge when triple interactions were included. Thus, we carried out independent analyses within groups, i.e., for each location and variety, we tested the effect of treatments and so on. For germination, analyses were carried out according to the seed and as the percentage of success of germination by subplot. When the main effects were significant, an LSD Fisher’s post hoc test was used (α = 0.05). All analyses were carried out using the IBM SPSS Statistics software package 23. An ANOVA test was carried out using the computer program STATGRAPHICS Centurion XVI software, version 16.1.03. No data transformations to meet ANOVA assumptions were necessary.

## 3. Results

### 3.1. Germination Percentage GP

This study assessed the effect of biostimulant application on the GP. The measurements were performed for each variety (AM, IM, and BS) and treatment (control, BioE, and BioE soil) under scrutiny, and measurements were taken at five DAS points: 17 DAS, 24 DAS, 33 DAS, 40 DAS, and 47 DAS in Plot A and 16 DAS, 23 DAS, 34 DAS, 41 DAS, and 48 DAS in Plot B. These results are shown in Table 2.

#### 3.1.1. Plot A over Time (DAS)

- 17 DAS: The BioE treatment demonstrates a positive impact on germination, apart from when administered to the seed in the BS variety. The IM variety shows the most effective response to BioE treatment in the soil, while AM exhibits the greatest responsiveness when BioE is applied to the seed.
- 24 DAS: Treatment with BioE promotes germination compared to the control in AM and IM while negatively affecting BS when biostimulant is applied to seeds and having no impact when applied to the soil. In addition, BioE applied to seeds shows a stronger response in AM. The best germination success for both AM and IM is achieved by adding BioE to the soil.
- 33 DAS: Germination has ceased in the control treatment, while seed germination persists in the two treatments with BioE. The detrimental effect of the BS treatment on seed disappears when compared to its control. The three varieties exhibit optimal responses when BioE is added to the soil, with AM showing the best response, closely followed by IM.
- 40 DAS: Very similar to 33 DAS, only continues the germination in AM control and AM e IM when the BioE is on the seed.
- 47 DAS: Germination has ceased in all of the plots and only continues in the AM control.

#### 3.1.2. Plot B over Time

- 16 DAS: The BioE treatment has a positive effect on the GP, except for the case of the IM variety treated with BioE on soil, which displays a lower germination rate than the control. The AM and BS varieties that were not treated do not show any germination. The BioE treatment on seed yields the most efficient response in IM, while both types of biostimulant applications elicit a similar response in AM and BS.
- 23 DAS: Treatment with BioE enhances germination compared to the control in AM and IM but has a lesser impact when applied to the soil. Moreover, BioE applied to seeds elicits a stronger response in AM. Optimal germination success for both AM and IM is achieved by adding BioE to the seed.
- 34 DAS: Germination has ceased in BS treated with BioE, whereas it continues in all other treatments and varieties. AM and IM show optimal responses when BioE is added to the seed, with IM exhibiting the highest response in all treatments, achieving 93.33% germination success.
- 41 DAS: The germination picture is like that of 33 DAS, with the germination continuing in the AM control.
- 48 DAS: Germination has ceased in almost all plots except for the AM and IM control plots, where it continues.

**Figure 2:**
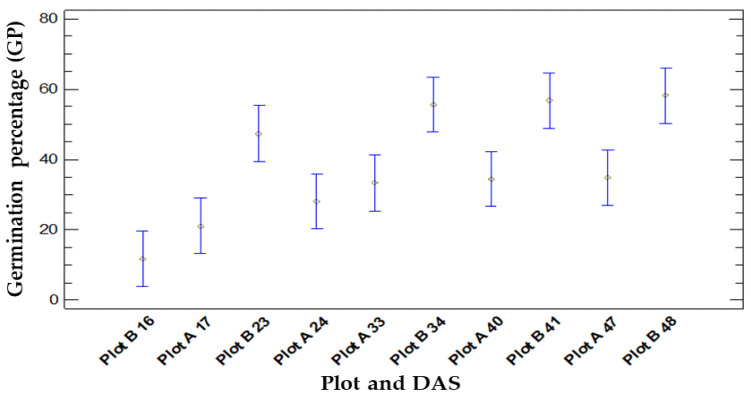
Values represent GP means with 95% Fisher’s LSD intervals obtained in Plot A and Plot B over time in the different DAS (days after sowing) analysed.

After the statistical analysis, the results obtained are represented in Figure 2.

When comparing GP means over time (DAS), we discovered a *p*-value of 0.0000, demonstrating significant differences with α = 0.05. The Fisher’s least significant difference (LSD) procedure was used in the multiple range test to distinguish between the means, which revealed statistically significant differences with a 95% confidence level among various samples, prompting the identification of four homogeneous groups. The initial significant divergence appears on the first day after sowing (16 DAS Plot A and 17 DAS Plot B). The subsequent divergence isolates the average germination ratios discovered in the remaining DAS of Plot A. The most noteworthy and meaningful germination ratios were established from the 34 DAS of Plot B.

There was a reduction in seed germination between 17 and 24 DAS in Plot A, whereas Plot B demonstrated a notable increase during 16 to 23 DAS (Figure 1).

#### 3.1.3. Comparison between Germination and Location (Plot A and B)

The GP means for Plot A are the lowest, averaging 30.37% from the 45 cases studied. On the other hand, Plot B has the highest mean germination, with an average of 45.93% from the 45 cases analysed. Furthermore, it has been observed that the mean GP of all varieties and treatments utilised is greater in Plot B than in Plot A, except for the AM variety treated with biostimulant on the soil (AM BioE soil), which showed a similar mean GP.

When we compared the germination success in the two locations, we found that the statistical analyses after the ANOVA test showed a value of *p* = 0.0005, which indicates that there are significant differences between Plot A and Plot B, with a significance level of 0.05. All of these results are graphically represented in Figure 2.

#### 3.1.4. Comparison between GP and Variety (AM, IM, and BS)

Figure 2 illustrates the variation in GP across different varieties. There is a notable dissimilarity observed in the BS variety when compared to the others. Statistical analysis reveals significant differences in GP based on the variety used (AM, IM, and BS). The results show statistically significant variations between the different varieties, with a *p*-value of 0.0000. Two homogeneous groups were identified using the multiple range test. The first group consists of the BS variety, which has an average GP of 23.33%, which is significantly lower than the second group comprising AM and IM, with averages of 44.45% and 46.67%, respectively.

#### 3.1.5. Comparison between GP and Treatment

**Figure 3:**
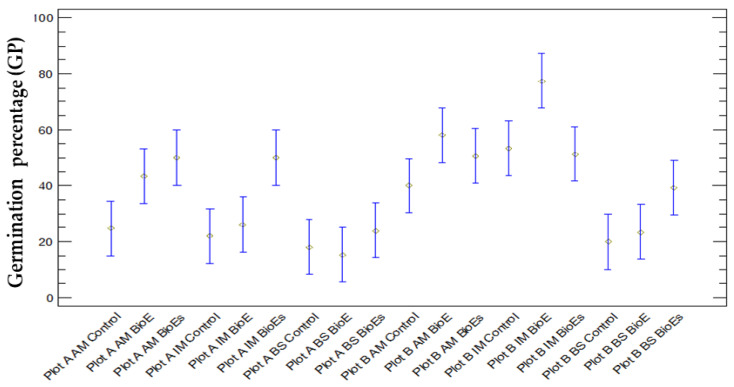
Means with 95% Fisher’s LSD intervals are presented for the GP values considering plot location (A and B), variety (AM, IM, and BS), and treatment (control, BioE, and BioEs).

When comparing GP across treatments (control, BioE, and BioE soil), statistically significant differences were found (*p* = 0.0258). Two homogeneous groups were identified in the multiple range tests, with their means discriminated using Fisher’s LSD method. The treatment control resulted in a 29.67% average in GP after the study of 30 cases, demonstrating significant differences compared to the treatment of the biostimulant applied directly to the soil (BioE soil), which had an average of 44.22%. However, the treatment with biostimulant on the seed (BioE), with an average of 40.56%, did not show statistical differences with either the control or the BioE soil (Figure 3).

#### 3.1.6. Comparison between GP and Location, Variety, and Treatment

1. Plot A:
  The application of a biostimulant treatment on the AM variety reveals a marked enhancement in the GP when compared to untreated controls. This improvement is evident when applied to either the soil or the seed, with BioE showing an average increase from 26.67 to 46.67 and up to 60 in BioE soil, leading to a 20% and 33.33% increase, respectively.
  For IM, the application of biostimulants to the seed was found to have a minimal effect on the GP compared to the untreated control. Nevertheless, the application of BioE to the soil resulted in a significant increase in germination, with the average increasing from 26.66 to 56.66, an increase of 30%.
  In contrast, there was only a small increase in the GP from 20 to 26.66% when BS was applied to the soil in comparison to the control. There was no effect when BS was applied to the seed.
2. Plot B:
  For the AM variety, the application of a biostimulant on the seed resulted in an enhanced GP compared to untreated seeds. Specifically, the GP rose by an average of 10%, from 60% in the control group to 70% in the BioE-treated group. Conversely, incorporation of the biostimulant directly into the soil did not cause any noticeable changes, as the GP remained unchanged compared to the control group.
  Treatment of IM with BioE increased the GP compared to the untreated control group, with an average rate of 73.33 increasing to 93.33, resulting in a 20% improvement in germination when the seed was coated with the biostimulant. However, BioE soil had a detrimental effect, as the mean GP dropped to 66.67, resulting in a 6.66% decrease relative to the control group.
  Regarding BS, only soil treatment led to a 20% increase in the GP from 26.67% to 46.67% compared to the control. No significant effect was observed when the biostimulant was applied to the seed (Figure 3).

When the mean GP was compared, considering all of the parameters analysed, such as location (A and B), variety (AM, IM, and BS), and treatment (control, BioE, and BioE soil) together (Figure 2), statistically significant differences were found (*p* = 0.0000), and six homogeneous groups were identified in the multiple range tests, with their means discriminated using Fisher’s LSD method. The analysis revealed 76 significant differences when comparing samples in pairs. The most significant difference with the lowest mean GP was Plot A, BS BioE, followed by Plot A, BS control. The samples with a higher mean GP were Plot B, IM BioE and Plot B, AM BioE.

**Figure 4:**
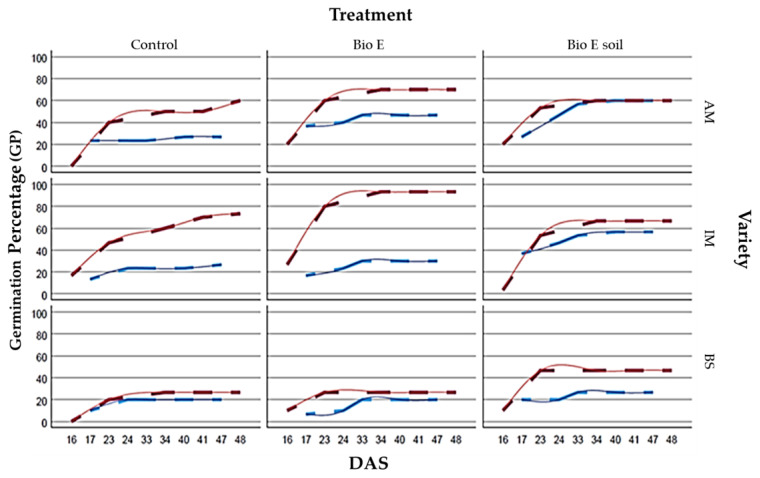
Values represent means of the GP for three different varieties, Amelia (AM), IMIDRA 10 (IM), and Blanco Sinaloa (BS), over time (DAS) under three biostimulant treatments (control, BioE, and BioE on soil). The blue line represents the result from Plot A, and the red line represents the result from Plot B.

If Plot A is taken into consideration, the treatments that showed the lowest germination success were the controls of the three varieties used (AM control, IM control, and BS control) and the BS variety when the biostimulant was applied to the soil (BS BioE soil). On the contrary, the greatest germination success is observed in the case of the IM variety treated with biostimulant applied on the soil (BioE soil) and AM treated with biostimulant in both seed and soil. In Plot B, the variety with the least successful germination is BS under both the control treatment and the biostimulant applied to the seed (BS control and BS BioE). Conversely, the varieties and treatments with the greatest germination success have been IM and AM, with the biostimulant applied to the seed (Figure 4).

### 3.2. Vegetative Development

This study assessed the effect of biostimulant application on the mean plant length (in cm) and node count. Ten plants were randomly selected for each treatment and variety under scrutiny, and measurements were taken at two DAS points (33 DAS and 40 DAS) in Plot A for both plant length and node count. Another plant length measurement was taken at DAS point 47. Plot B was utilised to assess the plant length and quantity of nodes at four diverse DAS points (23 DAS, 33 DAS, 41 DAS, and 48 DAS). The means and Standard Deviations (SDs) of measurements taken are reported in Table 3 for plant length (cm) and in Table 4 for the number of nodes.

#### 3.2.1. Average Plant Length

##### Comparison of Plant Length and Location (Plot A, Plot B)

When comparing the length (cm) of chickpea plants grown in Plot A and Plot B, statistical analysis reveals a significant difference (*p* = 0.0008), indicating that location affects plant growth. The average length of plants in Plot A was 13.3 ± 0.6 cm, while plants in Plot B measured 19.91 ± 0.48 cm. The results of the multiple range test indicate significant differences between the samples, with a 95% confidence level. 

##### Comparison of Plant Length (cm) and Variety

**Figure 5:**
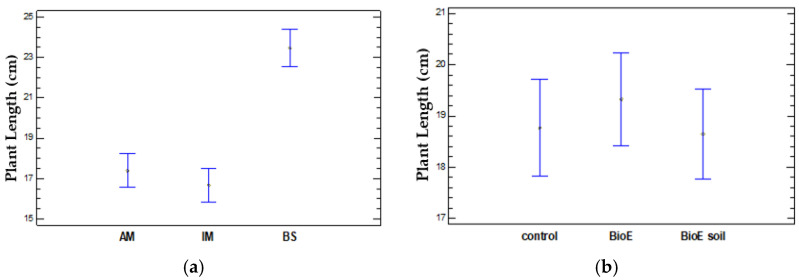
Mean values of the vegetative development of the measured plant length (cm) and 95% Fisher’s LSD intervals between the different varieties (AM, IM, and BS) (**a**) and treatments (control, BioE, and BioE soil) (**b**).

Significant differences appear (*p* = 0.0000) when comparing the plant length (cm) and variety (AM, IM, and BS). After conducting a multiple range test, two homogeneous groups were identified. Significant differences were seen at a 95% confidence level when comparing varieties BS with AM and BS with IM. There were no significant differences between the AM and IM varieties. Thus, one group will comprise the AM and IM varieties with an average plant length of, respectively, 17.4 ± 7.8 cm and 16.69 ± 10.56 cm. The other group will consist of the BS variety, which is longer on average at 23.45 ± 7.04 cm. These results are shown in Figure 5a. The BS variety displayed the highest length, while IM had the lowest, which was close to AM.

##### Comparison of Plant Length (cm) and Biostimulant Treatment

Upon comparing the mean plant lengths obtained for the different biostimulant treatments utilised (control, BioE, BioE soil). The ANOVA table shows a *p*-value of 0.7325 > 0.05, indicating that no statistically significant differences were found in plant length when biostimulants were applied, as is shown in Figure 5b.

##### Comparison of Plant Lengths concerning Location Treatment and Variety

An ANOVA table was prepared to compare the plant length (cm) of Plot A and Plot B, as well as the three varieties (AM, IM, and BS) and biostimulant treatments (control, BioE, and BioE soil). The results reveal statistically significant differences, with a confidence level of 95% and a *p*-value of 0.0000. To determine which variables differ, a multiple range test using Fisher’s LSD was performed. Consequently, 10 uniform clusters emerge along with 67 sets of samples that exhibit significant statistical variances.

It should be noted that the BS variety displays the most significant plant length development in both plots. In Plot B, the application of the biostimulant in both BioE and BioE soil forms demonstrates a favourable effect compared to the control, which is significant only between the BioE treatment and the control. In Plot A, the control in the BS variety achieved the greatest plant length. Although a negative effect was observed in the application of biostimulant, it was not significant for either the seeds or the soil. Nonetheless, it was still superior to the other varieties.

**Figure 6:**
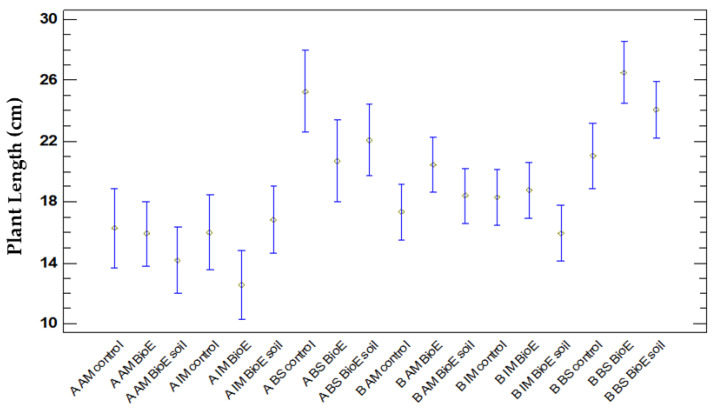
The values represent means of plant length, and 95% Fisher’s LSD intervals were calculated considering plot positions (A and B), varieties (AM, IM, and BS), and treatments (control, BioE, and BioE soil).

The AM and IM varieties in Plot A achieve significantly reduced plant length under all biostimulant treatments compared to their corresponding ones in Plot B. However, the BioE soil treatment in the IM variety resembles values in both plots. Figure 6 shows the results obtained after statistical studies.

**Figure 7:**
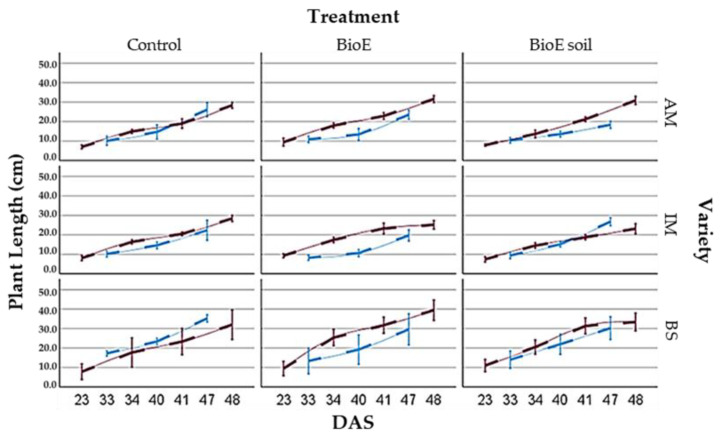
Graph of vegetative development. The values represent the mean plant length (cm) at different days after sowing (DAS) for different varieties and treatment and treatment in Plot A (in blue) and Plot B (in red).

In Plot A, negative effects are noted under biostimulant application in the AM variety, where chickpeas treated with BioE and BioE soil are shorter but insignificantly different from their control. In the IM variety, a negative effect is observed when the application is on seed, but it is not significant. In BS, this negative effect is also observed, but it is still not significant in both treatments. These facts have only been presented in Plot B of the IM variety when the biostimulant has been added to the soil (Figure 7).

#### 3.2.2. Average Number of Nodes

##### Comparison over Time (DAS)

**Figure 8:**
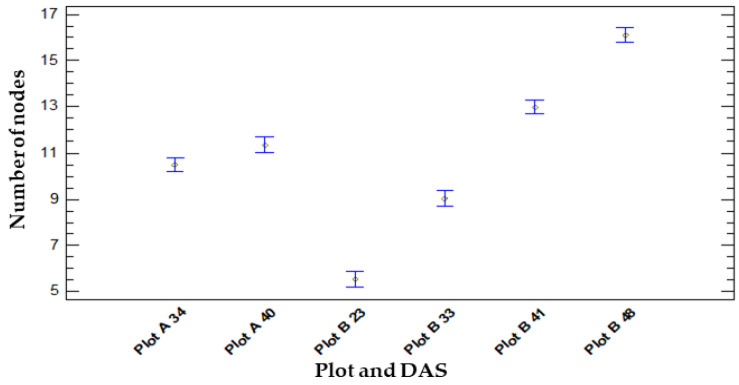
Graphical representation of vegetative development showing the average number of nodes and 95% Fisher’s LSD intervals on different DAS (days after sowing) and in different locations (Plot A. Plot B).

After 489 observations at different times (DAS) and locations (Plot A and Plot B), statistical analysis reveals significant differences between all DAS (*p* = 0.0000), as is graphically shown in Figure 8. 

##### Comparison of Number of Nodes and Location

When comparing the number of nodes on chickpea plants grown in Plot A and Plot B, statistical analysis indicates a significant difference (*p* = 0.0038), suggesting that location impacts plant growth. The mean number of nodes on plants in Plot A averaged 10.20 ± 2.28, while in Plot B it was 11.32 ± 4.38. The multiple range test was performed to analyse samples, revealing significant differences at a 95% confidence level. Plot B exhibited a more pronounced trend toward increased node numbers than Plot A, with a steeper gradient presented graphically in Figure 7.

##### Number of Nodes and Variety Comparison

**Figure 9:**
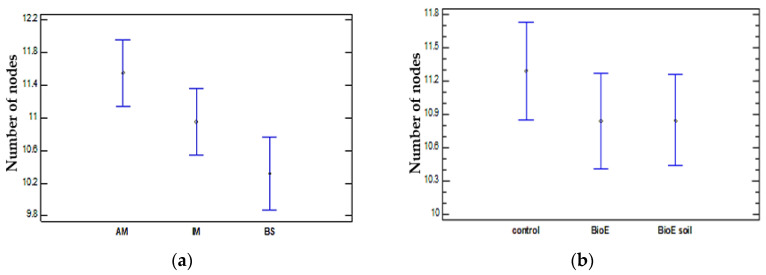
Mean values of the vegetative development of the number of nodes and 95% Fisher’s LSD intervals between the different varieties (AM, IM, and BS) (**a**) and treatments (control, BioE, and BioE soil (**b**).

Statistically significant differences (*p* = 0.0196) emerged when comparing the number of nodes in three varieties (AM, IM, and BS). After conducting a multiple range test, two groups with homogenous findings were identified. When comparing the BS and AM varieties, significant variations were detected at a 95% confidence level. This is illustrated in Figure 9a. The AM variety had a greater number of nodes compared to IM, with no statistical differences found. BS shows the lowest number of nodes and is statistically significant when compared with AM, and there are no differences when compared with IM.

##### Comparison of Number of Nodes and Biostimulant Treatment (Control, BioE, and BioE Soil)

The ANOVA table reveals that the number of nodes observed in different biostimulant treatments encompassing the control, BioE, and BioE soil demonstrate a *p*-value of 0.5028 (>0.05). As a result, the use of biostimulants does not exhibit any statistically significant impact on the number of plant nodes. This outcome is in line with the findings obtained for plant length. Figure 9b displays the node count and its 95% intervals. The biostimulant treatment has a non-significant negative impact on both seed and soil biostimulant application.

##### Comparison of Number of Nodes concerning Location, Treatment, and Variety

**Figure 10:**
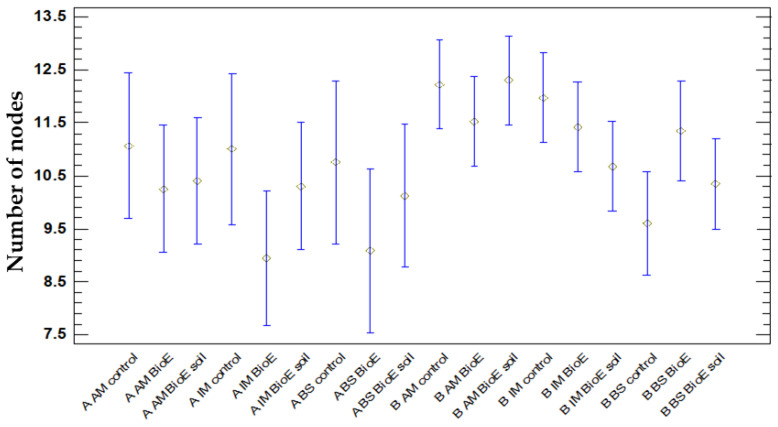
Average number of nodes and 95% Fisher’s LSD intervals were calculated considering plot positions (A and B), varieties (AM, IM, and BS), and treatments (control, BioE, and BioE soil).

An ANOVA table was prepared to compare the mean node number of Plot A and Plot B, as well as three varieties (AM, IM, and BS) and biostimulant treatments (control, BioE, and BioE soil) across a total of 489 observations. The results reveal statistically significant differences with a confidence level of 95% and a *p*-value of 0.0397. To determine which variables differ, a multiple range test using Fisher’s LSD was performed. Consequently, 5 uniform clusters emerge, along with 15 sets of samples that exhibit significant statistical variances. These statistical differences are graphically expressed in Figure 10.

The observed differences appear to be attributed to the location (Plot A or Plot B) and variety (AM, IM, or BS), whilst the type of treatment employed (control, BioE, or BioE soil), did not have a significant impact. 

**Figure 11:**
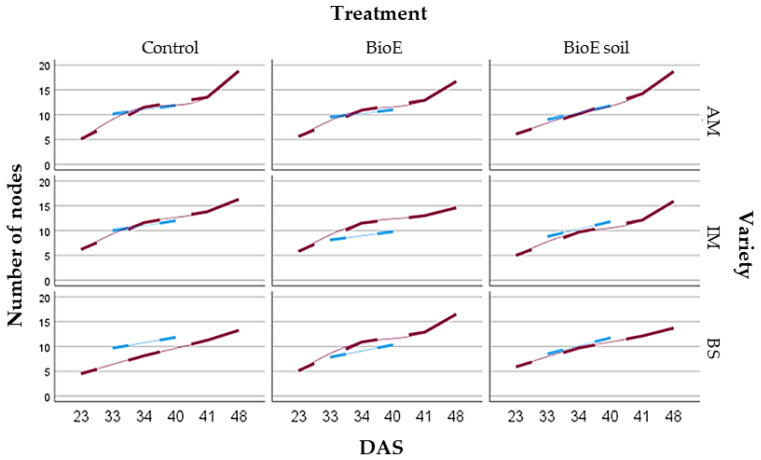
Vegetative development is measured as the means of number of nodes on different DAS (days after sowing) according to the variety (AM, IM, and BS) and treatment (control, BioE, and BioE soil) in Plot A (blue line) and Plot B (red line).

This aligns with the findings of the plant length measurements (cm), with the exception that the BS variety displayed the lowest number, while AM had the highest. The results are graphically represented in Figure 11.

The mean values for the nodes exhibit a small range, ranging from 8.9 to 12.3. It is noteworthy that when biostimulant is applied to both plots and varieties, it demonstrates a non-significant negative impact on the average number of nodes compared to the control. Only in the BS variant and in Plot B does it seem to be advantageous, but even then, there is no significant rise in the number of nodes.

### 3.3. Soil

The distinction in carbonate composition between the two soils is noteworthy, with 0.0% in Plot B and 6.05% in Plot A. The soils have been classified based on their composition (Table 1); Plot A is moderately alkaline and non-saline, with a very low phosphorus (P) concentration and optimal potassium (K) concentration. Plot B, on the other hand, is pH-neutral and non-saline, with an optimal concentration of phosphorus (P) and potassium (K), which is slightly higher than that of Plot A. Soil organic matter (SOM) is widely considered to be a crucial indicator of soil health, with both being of the same grade.

Siliceous-clay or silty-clay soils that are rich in potassium (K), and phosphorus (P) are optimal for chickpea cultivation. Although clay soils can also be used, their high clay content may lead to inferior-quality chickpeas. Additionally, soils with a high gypsum content should be avoided as they produce poor-quality chickpeas that are difficult to cook and of poor quality. The pH of the soil should be within 6 to 9, but acid soils should be avoided as they are more prone to *Fusarium* issues. Chickpeas are impacted by salinity and require well-aerated soil, as waterlogging can damage their growth [36]. 

**Figure 12:**
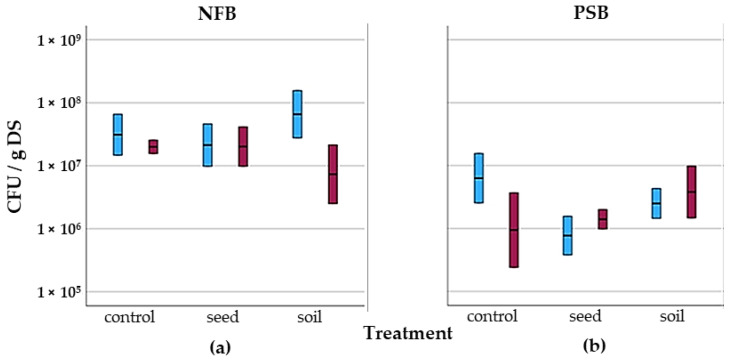
Nitrogen-fixing bacteria (NFB) (**a**) and phosphate-solubilising bacteria (PSB) (**b**) colony-forming units per gram of dry soil (CFU/g DS) following biostimulant application in seeds and soil. The blue bars represent Plot A, while the red bars represent Plot B. Bacterial concentration (CFU/g DS) is shown in the log scale.

Nitrogen (N) and phosphorus (P) are essential in the soil for the development of chickpea crops. However, nitrogen-fixing and phosphorus-mobilising bacteria are capable of increasing bioavailability in the soil. Figure 12 shows the results obtained from the measurements of both NFB (Figure 12a) and PSB (Figure 12b).

Nitrogen-fixing bacteria (NFB) counts remained in the same order of magnitude (10^7^–10^8^ CFU/g DS) for all treatments in Plot A (Figure 12a). In the case of Plot B, the BioE soil treatment shows a decrease in NFB counts (7.31 × 10^5^ CFU/g DS) compared to the other treatments and also between plots. As for phosphorus-solubilising bacteria (PSB), an average count of 10^6^–10^7^ CFU/ g DS was observed (Figure 12b). Although significant differences are observed in the control comparing plots, the PSB count in Plot B is lower (9.42 × 10^5^ CFU/g DS) than in Plot A (6.29 × 10^6^ CFU/g DS).

## 4. Discussion

In the current study, varying impacts of biostimulants have been observed in three processes, including germination, vegetative development, and the presence of NFB and PSB, as a measure of interaction with soil microorganisms. In previous studies conducted by our group [21], germination success was measured in the field. However, it was not possible to calculate the GP due to the difficulty in accurately determining the number of seeds sown using a seed drill. This study enabled quantitative calculation of the GP in open fields due to the experimental design, which allowed for a more precise evaluation of the biostimulant treatment on germination success. This study used a sample size of 10 individuals to calculate the mean vegetative development for each biostimulant dose, DAS, and variety, thus eliminating biases that may have been produced by a smaller sample size. The novelty of this trial compared with the previous one [21] lies in the fact that the germination success could be controlled by the total number of chickpeas sown in each subplot.

### 4.1. Germination Success

In the case of germination, which is a crucial step in the propagation of the plant and, ultimately, the yield of the crop, a clear positive effect was observed in contrast to vegetative development, where a non-significant negative effect was observed.

Concerning germination, some authors have also found this type of positive effect on germination and argue that the impact is dependent on the genotypes under investigation [37]. Conversely, some authors have reported adverse outcomes on seed germination due to application methods [5]. Specifically, previous studies performed by our group have shown that seeds that have been wetted and then dried (BioE) can result in a decreased GP [21]. Nevertheless, our present results revealed that this application method had a comparatively higher GP that did not significantly differ from the control. Significant discrepancies were noticed in comparison to the control when the biostimulant was directly administered to the soil in agreement with Li et al., (2022) [5]. This finding supports the notion that exposure to high volumes of biostimulant moisture before sowing reduces germination, in accordance with previous results found by our group [21].

A distinct variation in germination success has been observed depending on the site of the experiment, with superior results noted in Plot B in comparison to Plot A. This outcome may be attributed to the soil type found in Plot B, where the lack of carbonates is significant. The absence of carbonates in Plot B may, therefore, enhance the effects of the biostimulant applied to the soil, thus resulting in an increased GP, as is argued in [38]. Carbonates are recognised to have a significant influence on soil fertility and can result in reduced crop yields if they are present in excessive quantities [38].

Variations in germination success have also been observed among the studied varieties. Notably, the BS variety exhibits a significant difference when compared to the other two varieties (AM and IM), displaying a lower GP in both locations and across all three treatments according to the findings obtained by our group when the study was conducted in a greenhouse with different biostimulant and soil (substrate) conditions [21,27]. The other two cultivars, AM and IM, exhibited a comparable and markedly better reaction to germination, providing further evidence to the claims made by [37,38], who link the response to the biostimulant to the genotype of different varieties.

The analysis of variety, treatment, and location indicates that the BS variety demonstrated the lowest rates of germination. This variety is unsuited to the local climatic conditions and requires irrigation [36,39]. The AM and IM varieties demonstrated the most successful GP in both locations, and they were specifically selected for their superior adaptation to the local climatic conditions [20,21]. The efficacy of the biostimulant coating is particularly evident in Plot B when applied to AM and IM seeds, thus supporting the result of [37].

The biostimulant is composed of seaweed extracts, from which the presence of gibberellic acid and auxins, both phytohormones, can be attributed to its positive effect on germination. While auxins do not have a direct effect on germination, they support the biosynthesis of gibberellic acid, which in turn promotes germination by stimulating alpha amylase activity. Also, other authors [17,37] suggest that *Bacillus* species and *Pseudomonas* species can produce cyanide hydroxide gas, which in high concentrations can be toxic to seeds, thus leading to inhibition of germination. 

### 4.2. Vegetative Development

#### 4.2.1. Plant Length

After analysing the vegetative development in terms of plant length and location, we observed significant differences, the greatest being found in Plot B, which indicates that soils with less carbonate composition and phosphorus favour the growth of chickpea plants after biostimulant application [5]. 

Upon examining the variety, we found that there are no significant differences in the case of AM and IM in terms of growth in length, but there is a significant difference in the case of BS, which reaches the greatest average length in both plots and treatments; these results are similar to those obtained previously by our group under greenhouse conditions [27] and in soil.

As for the treatment used, no significant differences were found. The utilisation of BioE on seed appeared to have a favourable impact on its plant length response, albeit not significant. There appears to be a minimal adverse impact of biostimulant treatment on soil (BioEsoil), which is not statistically significant when compared to the control and is less than that seen with BioE treatment, which is also not significant. Previous research conducted by our group on the Amelia variety in open fields [21] did not reveal any significant differences in plant length after biostimulant application. It was assumed that the presence of weeds could be responsible for this effect, as their growth would be favoured by the biostimulant. To eliminate any biases in biostimulant application, all types of weeds were removed from each subplot. The data suggest that there were no significant differences between the biostimulant treatment and the control in Plot A for the plant length of the three varieties. However, there was a slight reduction in plant length means after treatment compared to the control, although this was not statistically significant. This outcome has already been reported [21,40]. Under uncontrolled conditions, in open-field soils, the inoculated PGPR biostimulant will compete with the soil microflora, and sometimes the positive effects are lost [17].

Significant differences are apparent when considering everything, with the BS variety achieving the greatest plant lengths in both Plots A and B. The greatest lengths in Plot A are attained with no biostimulant application (A BS control). while in Plot B they are reached by coating the BS seeds and applying it to the soil (B BS BioE and B BS BioE soil). Previous research has demonstrated that the biostimulant only increases plant growth in pot trials, but in open-field trials, as in our case, growth was lower in the presence of the biostimulant, suggesting a competitive effect of the biostimulant that favoured weed growth against chickpea [21]. To mitigate this issue, we sought to eliminate, as far as possible, all weeds growing in each plot throughout the trial, and we found negative effects in Plot A for the three varieties and positive effects in Plot B in the case of biostimulant application in the BS variety. Based on the information above, it is hypothesised that the varying effects on plant growth are a result of the composition of the biostimulant interacting with the soil, rather than any influence from weeds.

Other researchers using PGPR on chickpea sowing as a seed coating on sandy soil discovered significant improvements in agronomic traits, including plant height. They argued that a particular symbiotic relationship exists between the genotypes and the *Rhizobium* inoculum used and reported increases of up to 52% [41].

#### 4.2.2. Number of Nodes

Significant differences in vegetative development were observed when comparing the number of nodes and developmental progress over time (DAS) between the two locations under study. Plot A exhibited a lower slope than Plot B, which could be attributed to variations in soil type [5].

When comparing the number of nodes and varieties, a statistically significant distinction arises between the AM and BS varieties. The AM and IM varieties exhibit a greater number of nodes than the BS variety, the latter of which reaches the greatest plant length but not the greatest number of nodes, according to our previous results [21]. These distinctions mainly stem from the genotypic differences of each variety [37,42].

When comparing the number of nodes with the application of a biostimulant, statistically significant differences were not found. The control treatment had the highest number of nodes, followed by BioE soil and, finally, BioE. If the application of a biostimulant decreased the number of nodes, it was not significant, according to previous studies performed by our group [21].

### 4.3. Soil

Both Plot A and Plot B display an optimal pH range (6 to 9) for the growth of chickpeas [36]. There is a difference in the percentage of carbonates, which is 0% in Plot B. Carbonates can decrease crop yields in some soils by limiting the response to fertilisation and may even hinder the cultivation of particular agricultural species. Moderate alkaline soils reveal the highest potential response to biostimulant application according to [5]. Furthermore, soil salinity was strongly positively correlated with biostimulant effectiveness, and there was a negative trend between soil organic matter (SOM) and response after biostimulant application. Concerning soil P and K levels, biostimulants function better in poor soil deficient in P and K nutrients [38]. This could be a plausible justification for the superior outcome observed in Plot B concerning germination.

The application of BioE in the soil for Plot B was carried out with the same batch used for Plot A after 19 days in a molasses base derived from plant extracts and seaweed suspension. It is possible that having water activity in the preparation stimulated growth in the first application for Plot A and, by the time of the second application in the soil, the bacteria lost viability [40]. For this reason, there is a lower NFB count in Plot B (Figure 12a). In the case of PSB (Figure 12b), bacteria do not lose viability due to water dissolution over time given their ability to form spores [43]. PSB has an optimal growth at 32 °C and pH = 8, and it has an easily assimilable C concentration of 8 g/L [44], so it is interesting that in the experimental plots, an organic fertilizer derived from plant extracts and seaweed is applied to supply the C source in the soil–plant system. The pH is higher than ideal for PSB growth in the soil of these experimental plots (8.7), and the temperature is excessive (>25 °C). Therefore, if the soil is not bioaugmented with BioE, phosphorus-solubilising activity decreases, and the PSB ends up dying [45].

## 5. Conclusions

The application of biostimulants (BioE) to seeds led to a successful GP for both AM and IM varieties. The development was significant in Plot B, especially for the IM variety, compared to the control. The application of biostimulants to the soil has notably improved the GP for the AM and IM variants in Plot A. However, while a positive effect was observed for AM in Plot B, it was not significant, while a slightly negative effect was observed for IM. The response to the biostimulant during germination was not significant for the BS variety but was slightly noticeable when applied to the soil. The chickpea variety genotype appears to determine the response to biostimulant application.

For germination, we can accept the null hypothesis that the application of biostimulant has a beneficial effect on the germination of chickpea seeds in the varieties AM and IM. The effect of soil application is marginally better and noticeable in BS. 

There is an equivalent response to the application of the biostimulant on seed (BioE) and soil (BioE soil) in terms of vegetative development. This is reflected in the plant’s length and number of nodes, with no significant variations detected. Rejecting the null hypothesis proves that the biostimulant does not have an impact on vegetative growth. It is worth noting that variations have been found in this growth, which are more significant depending on the observed genotype. The AM and IM cultivars have a shorter plant length but a greater number of nodes than BS. 

Differences were observed in both germination and vegetative growth across different locations, with Plot B showing greater discrepancies than Plot A, possibly owing to differences in soil composition.

Nevertheless, it is recommended that biostimulant application studies be conducted in both greenhouse and open-field environments to avoid biases resulting from climatic conditions or substrate quality. This will allow for a more accurate assessment of the crops’ responses to the use of biostimulants in their respective environmental conditions.

## 6. Future Works

Our research group has been working with chickpeas for the last three years by applying biostimulants for agricultural improvement. However, we have experience in selecting optimal and resistant varieties for this area of central Spain since 2001.

As part of a research project associated with chickpeas, we discovered the potential of biostimulants for improving germination in open-field assays. This manuscript describes the investigation of how soil–plant and microbial interactions are affected by applying a biostimulant in two different ways depending on the initial richness and composition of the soils. While we acknowledge the shortness of the length of this study, we intended to obtain preliminary results that could be used to guide future long-term studies.

Therefore, we aim to share the obtained results to enable any researcher or farmer to replicate them and to continue with research on long-term assays that could provide more valuable information on chickpea cultivation.

## Figures and Tables

**Table 1:** Analytical values of soil samples from Plots A and B.

Plot A		Plot B	
Parameter	Value	Parameter	Value
pH	8.4	pH	7.2
C.E. (dS/m)	0.19	C.E. (dS/m)	0.14
Carbonates (%)	6.05	Carbonates (%)	0.00
N (%)	0.12	N (%)	0.12
SOM (%)	2.02	SOM (%)	2.02
P (mg/kg)	1.4	P (mg/kg)	67.9
Ca (mg/kg)	5159	Ca (mg/kg)	2113
Mg (mg/kg)	411	Mg (mg/kg)	415
Na (mg/kg)	34	Na (mg/kg)	105
K (mg/kg)	119	K (mg/kg)	227

**Table 2:** GP for different varieties (AM, IM, and BS) and treatments (control, BioE, and BioE soil) over various days after sowing (DAS) in both Plot A and Plot B.

Germination Percentage (GP)		Plot A					Plot B				
Variety	Treatment	17 DAS	24 DAS	33 DAS	40 DAS	47 DAS	16 DAS	23 DAS	34 DAS	41 DAS	48 DAS
AM	Control	23.33	23.33	23.33	26.67	26.67	0.00	40.00	50.00	50.00	60.00
	BioE	36.67	40.00	46.67	46.67	46.67	20.00	60.00	70.00	70.00	70.00
	BioE soil	26.67	46.67	56.67	60.00	60.00	20.00	53.33	60.00	60.00	60.00
IM	Control	13.33	23.33	23.33	23.33	26.67	16.67	46.67	60.00	70.00	73.33
	BioE	16.67	23.33	30.00	30.00	30.00	26.67	80.00	93.33	93.33	93.33
	BioE soil	36.67	46.67	53.33	56.67	56.67	3.33	53.33	66.67	66.67	66.67
BS	Control	10.00	20.00	20.00	20.00	20.00	0.00	20.00	26.67	26.67	26.67
	BioE	6.67	10.00	20.00	20.00	20.00	10.00	26.67	26.67	26.67	26.67
	BioE soil	20.00	20.00	26.67	26.67	26.67	10.00	46.67	46.67	46.67	46.67

**Table 3:** Means and Standard Deviations (SDs) of plant length (cm) for different treatments (control, BioE, and BioE soil) and varieties (AM, IM, and BS) on various days after sowing (DAS) in both Plot A and Plot B.

Plant Length (cm)		Plot A						Plot B							
		33 DAS		40 DAS		47 DAS		23 DAS		33 DAS		41 DAS		48 DAS	
Variety	Treatment	Mean	SD	Mean	SD	Mean	SD	Mean	SD	Mean	SD	Mean	SD	Mean	SD
AM	Control	10.1	2.6	14.0	4.3	26.1	3.5	7.0	1.4	14.9	1.6	19.0	3.4	28.4	2.2
	BioE	10.9	2.4	13.4	4.3	23.5	3.3	9.5	2.8	18.0	1.9	22.9	2.4	31.6	2.5
	BioE soil	10.4	2.0	13.8	2.2	18.4	2.6	7.9	0.8	13.7	2.8	21.2	1.8	30.9	2.9
IM	Control	10.2	1.7	14.6	2.0	22.3	6.2	8.1	1.9	16.2	1.3	20.5	1.5	28.4	2.2
	BioE	8.2	1.7	10.6	2.4	19.7	3.5	9.3	1.5	17.4	2.1	23.3	3.9	25.1	3.1
	BioE soil	9.4	2.4	15.1	2.1	26.8	2.8	7.4	2.0	14.5	2.1	18.7	1.9	23.2	3.7
BS	Control	17.3	1.4	23.4	1.7	35.2	1.8	7.9	3.9	17.7	9.0	23.3	8.1	32.0	9.1
	BioE	13.4	6.3	19.2	7.2	29.6	7.7	9.5	4.4	25.4	5.0	31.7	5.0	39.4	6.3
	BioE soil	14.0	5.4	22.0	6.3	30.2	7.1	11.0	4.4	20.5	5.1	31.4	5.8	33.4	6.4

**Table 4:** Means and Standard Deviations of the number of nodes for various treatments. Varieties: Amelia (AM), IMIDRA 10 (IM), and Blanco Sinaloa (BS) on different days after sowing (DAS) in both Plot A and Plot B.

Number of Nodes		Plot A				Plot B							
		33 DAS		40 DAS		23 DAS		33 DAS		41 DAS		48 DAS	
Variety	Treatment	Mean	SD	Mean	SD	Mean	SD	Mean	SD	Mean	SD	Mean	SD
AM	Control	10.14	1.35	11.88	2.85	5.10	1.10	11.50	0.97	13.50	1.84	18.80	1.32
	BioE	9.50	1.08	11.00	3.16	5.60	1.26	10.90	1.29	12.90	1.29	16.70	1.34
	BioE soil	9.00	1.49	11.80	1.32	6.10	0.57	10.20	2.04	14.20	1.12	18.70	1.79
IM	Control	10.00	1.15	12.00	0.82	6.20	0.79	11.60	0.70	13.80	0.79	16.30	1.70
	BioE	8.11	2.15	9.78	1.92	5.80	1.32	11.50	1.18	13.00	0.67	14.60	2.50
	BioE soil	8.80	1.75	11.80	1.03	5.00	1.05	9.70	1.42	12.10	1.29	15.90	1.73
BS	Control	9.67	0.82	11.83	1.17	4.50	2.26	8.13	4.09	11.25	3.01	13.25	3.33
	BioE	7.83	1.94	10.33	2.42	5.13	2.10	10.88	2.42	12.88	1.55	16.50	1.41
	BioE soil	8.50	2.78	11.75	2.76	5.90	1.52	9.70	2.16	12.10	2.85	13.70	2.36

## Data Availability

The raw data supporting the conclusions of this article will be made available by the authors on request.
